# Identification of novel *LEPR* mutations in Pakistani families with morbid childhood obesity

**DOI:** 10.1186/s12881-018-0710-x

**Published:** 2018-11-15

**Authors:** Robina Khan Niazi, Anette P Gjesing, Mette Hollensted, Christian Theil Have, Niels Grarup, Oluf Pedersen, Asmat Ullah, Gulbin Shahid, Wasim Ahmad, Asma Gul, Torben Hansen

**Affiliations:** 1 Department of Bioinformatics and Biotechnology, International Islamic University, Islamabad, Pakistan; 2 Novo Nordisk Foundation Center for Basic Metabolic Research, Section of Metabolic Genetics, Faculty of Health and Medical Sciences, University of Copenhagen, Copenhagen, Denmark; 3 Children Hospital, Pakistan Institute of Medical Sciences, Islamabad, Pakistan; 4 Department of Biochemistry, Faculty of Biological Sciences, Quaid-i-Azam University, Islamabad, Pakistan

**Keywords:** Early-onset obesity, Hyperphagia, Leptin, Leptin receptor, Melanocortin 4 receptor, Monogenic obesity, Pakistani families, Targeted resequencing

## Abstract

**Background:**

Mutations in the genes encoding leptin (*LEP*), the leptin receptor (*LEPR*), and the melanocortin 4 receptor (*MC4R*) are known to cause severe early-onset childhood obesity. The aim of the current study was to examine the prevalence of damaging *LEP, LEPR,* and *MC4R* mutations in Pakistani families having a recessive heritance of early-onset obesity.

**Methods:**

Using targeted resequencing, the presence of rare mutations in *LEP*, *LEPR*, and *MC4R*, was investigated in individuals from 25 families suspected of having autosomal recessive early-onset obesity. Segregation patterns of variants were assessed based on chip-based genotyping**.**

**Results:**

Homozygous *LEPR* variants were identified in two probands. One carried a deletion (c.3260AG) resulting in the frameshift mutation p.Ser1090Trpfs*6, and the second carried a substitution (c.2675C > G) resulting in the missense mutation p.Pro892Arg. Both mutations were located within regions of homozygosity shared only among affected individuals. Both probands displayed early-onset obesity, hyperphagia and diabetes. No mutations were found in *LEP* and *MC4R*.

**Conclusions:**

The current study highlights the implication of *LEPR* mutations in cases of severe early-onset obesity in consanguineous Pakistani families. Through targeted resequencing, we identified novel damaging mutations, and our approach may therefore be utilized in clinical testing or diagnosis of known forms of monogenic obesity with the aim of optimizing obesity treatment.

**Electronic supplementary material:**

The online version of this article (10.1186/s12881-018-0710-x) contains supplementary material, which is available to authorized users.

## Background

Currently, childhood obesity is considered one of the most serious public health challenges of the twenty-first century. The prevalence of childhood obesity is increasing at an alarming rate, affecting high-income as well as low- and middle-income countries, and the number of overweight and obese children below the age of five is estimated to be 41 million [http://www.who.int/topics/obesity/en.].

A strong genetic factor is evident in the etiology of obesity, with heritability estimates ranging from 40 to 70% [1–3]. Genetic defects disrupting the leptin-melanocortin signaling pathway very often result in severe early-onset obesity and hyperphagia [4–6], and the genes most commonly involved in monogenic forms of obesity are part of this pathway, including *leptin* (*LEP*), the *leptin receptor* (*LEPR*), and the *melanocortin 4 receptor (MC4R*) [7–9]. Leptin is a 16-KD hormone secreted by white adipocytes which binds to LEPR and regulates energy expenditure through hypothalamic neurons [10]. LEPR is a member of the cytokine receptor family with six isoforms (LEPRa-f), yet, leptin signaling is primarily mediated by the long LEPRb expressed in the hypothalamus [11–13]. The short form is expressed in a number of tissues, including the adrenal gland, kidney, lung, and choroid plexus [14, 15]. The mutations identified to date in *LEP* and *LEPR* are ethnic-specific, and the prevalence of monogenic obesity caused by mutations within these two genes is as high as > 20% in Pakistani study populations with obesity [16–18].

*MC4R* encodes a 332- amino acid membrane-bound receptor protein [19]. It is expressed in the brain and it influences appetite regulation through interaction with adrenocorticotropic and melanocyte stimulating hormones (MSH) through G proteins [20]. In studies of European populations with severe obesity, the prevalence of heterozygous damaging *MC4R* mutations is ~ 6% and ~ 2% in children and adults, respectively [21–23], signifying the different etiologies of childhood and adult obesity.

The disruption of *LEPR* and leptin deficiency results in severe early-onset hyperphagic obesity with rapid weight increase during the first few months of life [17, 24, 25]. Similarly, during the first year of life, MC4R deficiency has been linked with hyperphagia and increased fat and lean mass, increased linear height, increased bone mineral density and severe early hyperinsulinemia [22, 26], although some of these associations remain controversial [27, 28]. Obesity caused by damaging mutations in *LEP* and *LEPR* display an autosomal recessive mode of inheritance while obesity caused by *MC4R* mutations exhibit variable penetrance (recessive or co-dominant) [23].

Genetic sequencing of consanguineous families is an important tool in the identification of deleterious mutations in genes implicated in monogenic forms of obesity [18], as consanguineous families share homozygous regions in their genomes, enabling the identification of deleterious recessive mutations [29]. Due to the high degree of inbreeding in the Pakistani population, homozygous deleterious mutations in *LEP, LEPR,* and *MC4R* have been identified in as many as ~ 30% of severely obese individuals from consanguineous families [16–18]. In comparison, the prevalence of heterozygous *MC4R* mutations is 3–5% in Caucasian populations with early-onset obesity [30, 31]. Both *LEP* and *LEPR* mutations are rare in Caucasian populations.

The application of next generation sequencing (NGS) provides a powerful method to discover rare disease-causing genetic variants [32]. Therefore, targeted resequencing of *LEP, LEPR* and *MC4R* was performed to assess the prevalence of damaging mutations within these genes. In total, genetic screening was performed in 25 severely obese probands from Pakistani families of which 14 probands were from families with known consanguineous marriages.

## Methods

### Study design

This family-based study applied a phenotype-driven approach to investigate monogenic causes of obesity in selected Pakistani families with obesity segregating in an autosomal recessive pattern.

### Participants

Twenty-five families, originating from different regions of Pakistan, were recruited and examined at Children Hospital, Pakistan Institute of Medical Sciences (PIMS), Islamabad. Consanguineous marriages were known in 14 of the included families. The selection of the families was based on three criteria: 1) Body mass index (BMI) of probands > 30 kg/m^2^; 2) Probands displaying obesity onset before five years of age; and 3) Parents of the probands with BMI < 25 kg/m^2^, consistent with an autosomal recessive mode of inheritance.

### Clinical examination

Through interview sessions, information on age of obesity onset (years), other major chronic disease(s) (if any), metabolic disorder(s) running in the family, eating habits, physical activity, along with obesity-related co-morbidities was recorded. Waist circumference (cm) and height (cm) were measured with a non-elastic plastic tape with the participant standing in an upright position without shoes. Weight (kg), without shoes and in light clothes, was measured to the nearest 0.1 kg using a digital scale. From these measures, BMI was calculated as the weight in kilograms divided by the square of the height in meters (kg/m^2^), and using the LMS method [33], a BMI standard deviation score (SDS) was calculated based on a World Health Organization (WHO) reference population [34]. Approximately 3–5 ml of venous non-fasting blood from affected and un-affected family members were collected in 8.5 ml vacutainer tubes (BD Vacutainer® ACD, Franklin Lakes NJ, USA). Clinical characteristics of the families are presented in Additional file 1.

### Genomic DNA extraction

DNA was extracted from blood samples from 36 affected and 88 unaffected family members. Genomic DNA was primarily extracted using the standard phenol-chloroform method [35], however, in some families, the QIAamp DNA Mini Kit (Qiagen, Germany) was used.

### Targeted resequencing

The probands (*n* = 25) from each family as well as four additional affected individuals (OB2–6, OB4–8, OB4–9, and OB4–10) underwent targeted resequencing. Using a chip-based customized nucleotide probe, targeted resequencing was performed to examine the coding regions of *LEP, LEPR,* and *MC4R.* Methods for target region capture and NGS have previously been extensively described [36]. According to the manufacturer’s standard cluster generation and sequencing protocols, the final captured DNA libraries were sequenced using the Illumina HiSeq2000 Analyzers as PE 90 bp reads. Only variants having a minimum mean depth of 20x were included. Identified variants were annotated according to the transcripts 1) *LEP*: NM_000230; 2) *LEPR*: NM_002303.5 and 3) *MC4R*: NM_005912.

### Chip genotyping

Illumina Infinium Human CoreExome Bead Chip (CoreExomeChip) genotyping was performed in 124 individuals from 25 families using Illumina’s HiScan system at the laboratory facilities of the Novo Nordisk Foundation Center for Basic Metabolic Research at Symbion, Copenhagen, Denmark. The standard pipeline in Illumina Genome Studio software was used for the genotype calling. The pipeline yielded 551,839 genetic variations, which entered our quality control (QC) pipeline. The Illumina final report was converted to plink format using custom scripts and aligned with the positive strand of the GRCh37 reference.

QC included removal of variants with missing genotypes above 5% as well as individuals with more than 5% missing genotype calls, individuals with negative inbreeding, indication of duplicated samples, and discrepancy between genetic identified pedigree and the pedigree obtained from the family. A total of 12 individuals were removed in the QC. We did not remove SNPs deviating from Hardy-Weinberg equilibrium as is otherwise usual, since these variants may be of interest given the specific mode of data sampling.

### Homozygosity mapping

Based on genotyping, runs of homozygosity were determined in each family using the “homozyg” command in PLINK [37].

## Results

In the present study, targeted resequencing data was combined with chip-based genotyping, enabling the identification of rare and potentially novel causal variants co-segregating with obesity. No coding variants were identified within *MC4R* and *LEP*. However, in *LEPR* eight coding variants were identified, of which two were synonymous, five were missense and one was a frame-shift mutation. Identified mutations were classified as potentially damaging, if they were: 1) non-synonymous; 2) rare in the general population with a minor allele frequency below 0.1% [38, 39] and 3) homozygous or if two or more heterozygous mutation were present within the same gene. Two identified variants fulfilled these criteria; one was a frame-shift variant (p.Ser1090Trpfs*6) and the other was a missense mutation (p.Pro892Arg). The pathogenicity of the missense variant was assessed using CADD scores [40].

### Frameshift mutation (Ser1090Trpfs*6)

**Fig. 1:**
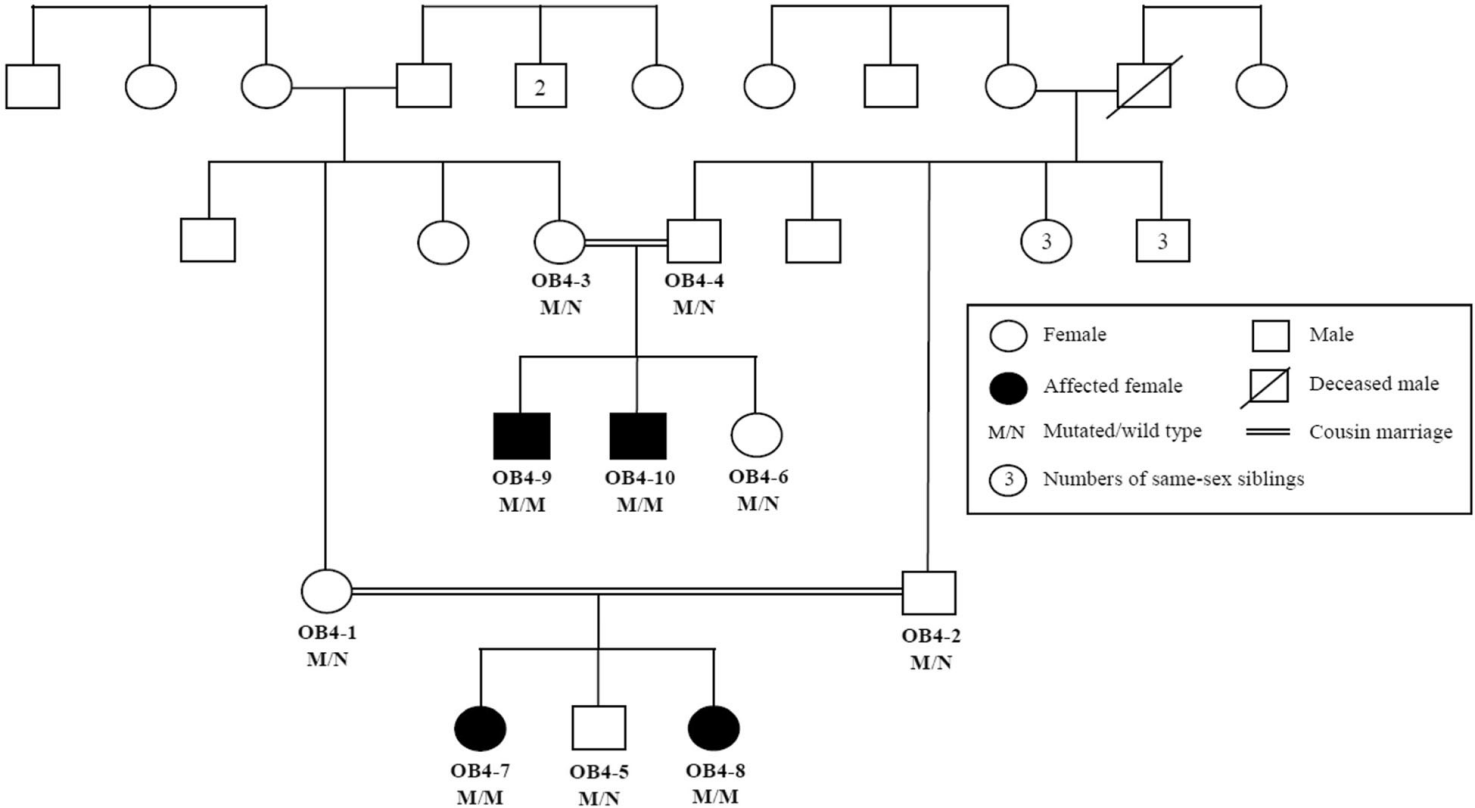
Pedigree of family OB4 with the frameshift mutation c.3260AG (p.Ser1090Trpfs*6) in *LEPR*

In family OB4, the novel homozygous frameshift mutation c.3260AG (p.Ser1090Trpfs*6) which truncates the LEPR protein, was identified in exon 20 of the *LEPR* (Fig. 1). This frameshift was the result of a deletion of AG at nucleotide position 66,102,459 to 66,102,461. This mutation was located within the homozygosity region on chromosome 1p31.1 from position 55,397,406 to 75,241,971. This homozygous region was only shared among affected family members.

Affected members in the family OB4, originating from the Rajanpur district of the Punjab province in Pakistan, had a very homogenous phenotype, comprising hyperphagia and rapid weight gain resulting in morbid obesity at an early age. This severe phenotype was evident in several affected family members, including two females (OB4–7 and OB4–8) aged 12 and 8 years, respectively, as well as two male first cousins (OB4–9 and OB4–10) aged 10 and 8 years, respectively. At the time of recruitment of the family, the proband OB4–7, aged 12 years, weighed 145.0 kg with a BMI SDS of 4.48 and BMI of 62.8 kg/m^2^ (Table 1). At follow-up, the proband was 14 years old with pubertal stage of Tanner IV [41], yet, without menarche and had developed diabetes with C-peptide level of 0.92 nmol/L. Moreover, the sibling (OB4–8) and two affected cousins (OB4–9, OB4–10) were in the pubertal stage of Tanner I [41, 42]. The severity of the mutation was evaluated based on the sex of the carriers, as a sex-specific effect of *LEPR* mutations has previously been suggested [43]. However, we did not find any effect of the mutation influenced by the sex of the carriers (Table 2).

**Table 1:** Clinical characteristics of probands with homozygous *LEPR* mutations

	Proband OB4–7	Proband OB25–4
Family ID	OB4	OB25
Sex	Female	Female
Age at enrolment (years)	12.18	1.03
Age at obesity onset	40 days	60 days
Height (cm)	152.0	76.2
Weight (kg)	145.0	18.0
BMI (kg/m^2^)	62.8	31.0
BMI SDS	4.49	6.49
Waist circumference (cm)	137.0	73.6
Family history of obesity	No	No
Related co-morbidities	Diabetes	Diabetes, dyslipidaemia, hepatic and renal function disorder
Mutation type	Frameshift	Missense

**Table 2:** Sex stratified analysis of BMI, weight and waist of homozygous carriers of the *LEPR* p.Ser1090Trpfs*6 mutation in family OB4

	BMI (kg/m^2^)	BMI SDS	Weight (kg)	Waist (cm)
Female carriers				
OB4–7	62.8	4.49	145.0	137.0
OB4–8	38.2	4.40	75.0	111.7
Mean (SD)	50.5 (17.4)	4.44 (0.062)	110.0 (49.5)	124.4 (17.9)
Male carriers				
OB4–9	40.8	4.18	80.2	111.7
OB4–10	41.0	5.39	55.0	99.0
Mean (SD)	40.9 (0.15)	4.79 (0.85)	67.6 (17.8)	105.4 (8.98)
*p*-value*	0.6	0.7	0.4	0.3

*Evaluated using a t-test

### Missense mutation (p.Pro892Arg)

**Fig. 2:**
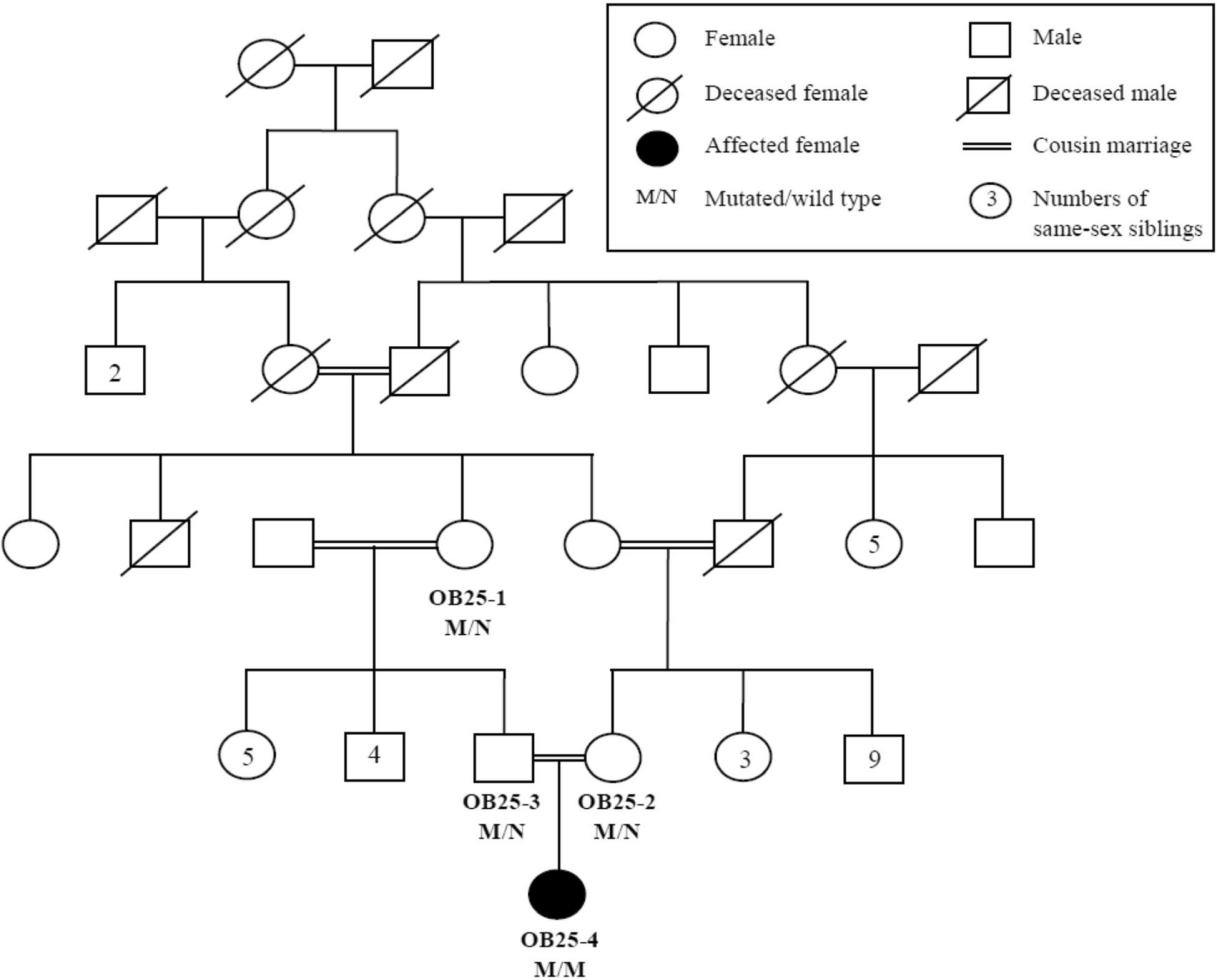
Pedigree of family OB25 with the missense mutation c.2675C > G (p.Pro892Arg) in *LEPR*

The novel missense mutation p.Pro892Arg, located in exon 20 in *LEPR*, was identified in proband OB25–4 of family OB25 (Fig. 2). This mutation was located at position 66,101,875 within a homozygous region from position 59,653,630 to 91,206,170 on chromosome 1p31. Born of a consanguineous marriage, the proband presented with a normal birth weight of 3.0 kg but after two months, she rapidly gained weight and attained the weight of 18.0 kg at the age of one year, corresponding to a BMI SDS of 6.49 (Table 1). In addition, the proband suffered from diabetes and displayed developmental delay. The proband was also in the pubertal stage of Tanner I. When annotated using online bioinformatics tools, p.Pro892Arg was predicted to be damaging and had CADD score of 28, strongly suggesting the mutation to be deleterious.

## Discussion

In the present study, we employed targeted resequencing as a means to identify the genetic etiology in cases with severe early-onset obesity. The sequencing data was analyzed with respect to the three main genes known to be involved in monogenic forms of obesity, i.e. *LEP, LEPR* and *MC4R.* Given the high prevalence of *LEP*, *LEPR* and *MC4R* mutations previously reported in Pakistani populations [18], a similarly high prevalence of causal variants within these genes was expected. However, only two of the 25 examined probands carried homozygous recessive mutations, and both mutations were positioned in *LEPR*.

*LEPR* mutations have previously been reported to influence the risk of developing severe early-onset obesity, hypogonadotropic hypogonadism and hypothalamic hypothyroidism [24, 44], which is similar to the clinical characteristics of leptin deficiency [24]. Furthermore, in mice, *Lepr* mutations have been found to influence the susceptibility of type 2 diabetes [45]. Hypogonadotropic hypogonadism in LEPR-deficient individuals may be due to a defect both at the hypothalamic and the pituitary level [46]. However, hypogonadism may change over time, as in the case of spontaneous pubertal development and a natural pregnancy [24].

Both of the novel *LEPR* mutations identified in the current study, are positioned in the intracellular domain of LepRb, which is involved in energy homeostasis, glucose metabolism, fertility, growth and the action of insulin [47, 48] . Upon binding, leptin activates the LepRb through the mediation of multiple signaling pathways, including phosphorylation of cytoplasmic tyrosine kinases of Janus Kinase 2 (JAK2), conscription of signal transducer and activator of transcription 3 (STAT3) and mitogen-activated protein kinase (MAPK) cascade [47, 49–51]. In both humans and mice, multiple forms of LepRb are known, including short intracellular domain forms ranging from 32 to 40 amino acids and the long form comprising 303 amino acids, which is predominantly expressed in the hypothalamus [14, 48, 52–54]. The missense mutation (p.Pro892Arg), identified in a family OB25, is located in the Box 1 motif which is important both for leptin-dependent JAK2 activation through the mediation of signaling by the intracellular domain and for the physiologic actions of leptin [52, 55]. The CADD score of the identified mutation (p.Pro892Arg) is 28, which indicates this is a very likely disease-causing variant. The second mutation, i.e. frameshift (Ser1090Trpfs*6) identified in the family OB4, is located in the long intracellular domain of LepRb and has sequence motifs resulting in the truncation of the domain, thereby suggesting a dysfunctional effect on its intracellular signal-transducing capabilities.

The highly deleterious nature of the identified two mutations (p.Pro892Arg and Ser1090Trpfs*6) is consistent with the clinical conditions of hyperphagia, rapid weight gain and extreme obesity observed in proband OB4–7. In both probands, the mutations were found in a homozygous state, and based on genotyping of family members, the homozygous region was found to be shared among affected individuals only, while parents of the probands were heterozygous carriers. Thus, when combined, our results strongly indicate that the identified mutations are causal.

Previously, it has been observed that boys in the Pakistani population are more prone to obesity than girls [56]. In addition, Iranian consanguineous families have revealed that LEPR deficiency may be more severe in females compared to males [43]. However, in OB4 where the mutation was found in both affected boys and girls, it showed the same level of severity irrespective of sex.

Increasing knowledge of genetic factors involved in the development of childhood obesity leads towards an improved understanding of the genetic etiology of this disorder. For this purpose the Pakistani population is unique due to its large size, its high number of families with known consanguineous marriages and the high frequency of large pedigrees [56]. Especially the identification of rare, damaging variants predisposing to obesity holds promise to the future development of novel therapeutic options and personalized medicine based on molecular diagnosis [24, 57]. In the case of congenital leptin deficiency caused by deleterious *LEP* mutations, hormonal leptin therapy has proved to have dramatic treatment effects, successfully decreasing the body weight and hyperphagia of the carriers [58, 59]. Recently, treatment with mechanism-based therapy using a MC4R agonist (setmelanotide) in two patients with damaging *pro-opiomelanocortin (POMC)* mutations completely reversed hyperphagia and induced a remarkable weight loss while normalizing insulin sensitivity [60]. Albeit no effective drug therapies are currently available for LEPR deficient individuals, treatment of dysfunctional POMC with MC4R-agonist suggests its efficacy in other monogenic defects of the hypothalamic leptin-melanocortin pathway, including *LEPR* deficient patients [60]. Hence, the treatment with setmelanotide might be an effective in treatment of probands with non-functional LEPR, as identified in our study.

Yet, within 23 of the probands included in the present study, no causal variants were identified. For these remaining probands, the application of whole exome sequencing may be an important means to examine whether damaging mutations in other, yet unknown genes, may be the cause of their inherited early-onset obesity.

## Conclusion

Using targeted resequencing in consanguineous Pakistani families, two novel mutations, including a frameshift and a missense mutation, were identified in probands with severe early-onset obesity. Both of these mutations were identified in a homozygous state. Our findings demonstrate the effectiveness of targeted resequencing to identify rare coding pathogenic mutations in consanguineous cases of severe early-onset obesity.

## Additional file

Additional file 1: Clinical characteristics of 34 affected individuals in 25 families. (DOCX 33 kb)

## Abbreviations

BMI: Body mass index
CADD: Combined annotation dependent depletion
IIUI: International Islamic University Islamabad
JAK2: Janus Kinase 2
LEP: Leptin
LEPR: Leptin-receptor
MAPK: Mitogen-activated protein kinase
MC4R: Melanocortin 4 receptor
MSH: Melanocyte stimulating hormone
NGS: Next generation sequencing
OB: Obesity
PCA: Principal component analysis
PIMS: Pakistan Institute of Medical Sciences
POMC: Pro-opiomelanocortin
QC: Quality control
SDS: Standard deviation score
SNP: Single nucleotide polymorphism
STAT3: Signal transducer and activator of transcription 3
