# Supplementary material for: Identification of novel LEPR mutations in Pakistani families with morbid childhood obesity
Source: BMC Med Genet. 2018 Nov 15;19:199. doi: 10.1186/s12881-018-0710-x (PMC6238292; doi:10.1186/s12881-018-0710-x)
Supplement: Supplementary file 1 — Clinical characteristics of 34 affected individuals in 25 families. (DOCX 33 kb) [file 12881_2018_710_MOESM1_ESM.docx]

| **Family ID** | **Subject ID** | **Sex** | **Age at enrolment (years)** | **Age at obesity onset (years)** | **Height (cm)** | **Weight**  **(kg)** | **BMI (kg/m^2^)** | **BMI SDS** | **Waist circumference (cm)** | **Family history of obesity** | **Obesity-related co-morbidities** | **Family-related disorders** |
| --- | --- | --- | --- | --- | --- | --- | --- | --- | --- | --- | --- | --- |
| OB1 | OB1-4 | Male | 17.74 | <5 | 164.5 | 96.1 | 35.5 | 3.02 | N/A | No | Hyperphagia, hypertension | Hypertension |
|  | OB1-5 | Female | 9.93 | < 5 | 121.9 | 98.0 | 66.0 | 4.78 | N/A |  | Hyperphagia |  |
| OB2 | OB2-5 | Female | 23.14 | < 5 | 154.9 | 97.0 | 40.4 | 3.67 | 111.7 | No | Hyperphagia, hypertension | Hypertension |
|  | OB2-6 | Male | 9.50 | <5 | 111.8 | 40.0 | 32.0 | 3.92 | 81.2 |  | Hyperphagia |  |
| OB3 | OB3-4 | Male | 30.10 | < 5 | 173.7 | 99.3 | 32.9 | 1.03 | 106.6 | Yes | Hyperphagia, hypertension | None |
|  | OB3-5 | Male | 12.26 | < 5 | 143.2 | 78.1 | 38.1 | 3.61 | 96.5 |  | Hyperphagia |  |
|  | OB3-6 | Female | 10.02 | < 5 | 137.1 | 81.9 | 43.6 | 4.16 | 101.6 |  |  |  |
| OB4 | OB4-8 | Female | 8.29 | 40 days | 140.2 | 75.0 | 38.2 | 4.40 | 111.7 | No | Hyperphagia | None |
|  | OB4-9 | Male | 10.39 | 40 days | 140.2 | 80.2 | 40.8 | 4.18 | 111.7 |  |  |  |
|  | OB4-10 | Male | 8.03 | 40 days | 115.8 | 55.0 | 41.0 | 5.39 | 99.0 |  |  |  |
| OB5 | OB5-5 | Female | 12.28 | < 3 | 137.1 | 75.2 | 40.0 | 3.63 | 91.4 | Yes | Hyperphagia, hypertension | Diabetes, hypertension, asthma, heart disease |
| OB6 | OB6-5 | Female | 17.03 | < 3 | 152.4 | 67.1 | 28.9 | 1.93 | 86.3 | Yes | None | None |
|  | OB6-6 | Male | 14.94 | < 5 | 164.5 | 95.3 | 35.2 | 3.12 | 106.6 |  | Hyperphagia |  |
| OB7 | OB7-3 | Male | 15.12 | < 5 | 162.5 | 77.0 | 29.2 | 2.36 | 101.6 | Yes | Hypertension, gynaecomastia | Diabetes, hypertension, heart disease, nephropathy |
| OB8 | OB8-3 | Female | 6.70 | < 5 | 101.6 | 32.0 | 31.0 | 4.48 | 78.7 | Yes | Dyslipidaemia | Diabetes, hypertension |
| OB9 | OB9-5 | Male | 23.36 | < 5 | 177.8 | 132.0 | 41.8 | 3.11 | 111.7 | Yes | Hyperphagia, hypertension | Diabetes, hypertension, asthma |
| OB10 | OB10-4 | Female | 22.77 | ~ 5 | 162.5 | 81.0 | 30.7 | 2.10 | 111.7 | No | Polycystic ovary, hypertension | Cardiovascular disease, hypertension |
| OB11 | OB11-5 | Male | 16.95 | < 5 | 172.7 | 108.0 | 36.2 | 3.12 | 111.7 | Yes | Hyperphagia | Diabetes, hypertension |
| OB12 | OB12-3 | Male | 29.92 | ~ 5 | 170.2 | 115.0 | 39.7 | 2.03 | 114.3 | Yes | Diabetes, hypertension | Diabetes, hypertension, Osteoporosis, arthritis |
|  | OB12-4 | Male | 27.19 |  | 162.5 | 104.0 | 39.4 | 2.32 | 106.6 |  | Gout, ulcer, epilepsy, hypertension |  |
| OB13 | OB13-6 | Male | 13.21 | < 5 | 154.9 | 70.1 | 29.2 | 2.68 | 99.0 | Yes | Hypertension, nephropathy, chronic fatigue | Asthma |
| OB14 | OB14-5 | Female | 13.69 | < 5 | 157.5 | 80.3 | 32.4 | 2.78 | 109.2 | Yes | Hyperphagia | Diabetes, hypertension |
|  | OB14-6 | Male | 26.08 | ~ 5 | 177.8 | 115.0 | 36.4 | 2.07 | 124.4 |  | Hypertension |  |
|  | OB14-7 | Male | 19.28 |  | 172.7 | 105.0 | 35.2 | 2.91 | 119.3 |  | Hypertension |  |
| OB15 | OB15-5 | Male | 16.29 | ~ 2 | 152.4 | 76.0 | 32.7 | 2.73 | 104.1 | No | Developmental delay, CVD | None |
| OB16 | OB16-4 | Male | 13.11 | ~ 2 | 162.5 | 66.0 | 25.0 | 2.02 | 86.3 | Yes | Hyperphagia | Diabetes |
| OB17 | OB17-3 | Female | 0.66 | 3 months | 76.2 | 20.0 | - | 7.37 | 58.4 | No | Hyperphagia | None |
| OB18 | OB18-3 | Female | 23.33 | < 5 | 162.5 | 89.0 | 33.7 | 2.64 | 109.2 | Yes | Hypertension | Diabetes, hypertension, heart disease, nephropathy |
| OB19 | OB19-4 | Male | 15.32 | < 5 | 139.7 | 92.1 | 47.2 | 3.93 | 106.6 | No | Hyperphagia | Hypertension |
| OB20 | OB20-4 | Male | 19.03 | ~ 5 | 162.5 | 82.0 | 31.1 | 2.26 | 104.1 | No | Hyperphagia | None |
| OB21 | OB21-4 | Male | 19.95 | < 5 | 142.2 | 85.0 | 42.0 | 3.66 | 114.3 | Yes | Heart problem, hyperphagia | None |
| OB22 | OB22-3 | Male | 20.19 | ~ 5 | 177.8 | 122.0 | 38.6 | 3.22 | 119.3 | No | Hypertension | None |
| OB23 | OB23-5 | Male | 30.11 | ~ 5 | 177.8 | 115.0 | 36.4 | 1.57 | 124.4 | Yes | Hypertension, varicose vein problem | Hypertension, diabetes, nephropathy, metabolic syndrome |
| OB24 | OB24-10 | Male | 11.18 | 1.5 | 137.1 | 53.0 | 28.2 | 2.98 | 96.5 | No | Hyperphagia, fatigue, continuous head movement, weak eye sight | Nephropathy, asthma, cardiovascular disease, arthritis |
